# Creatine monohydrate for lean mass, strength, and bone density in postmenopausal women: a systematic review and meta-analysis

**DOI:** 10.1080/15502783.2026.2668435

**Published:** 2026-05-16

**Authors:** Siavash Naddafha, Jose Antonio, Richard B. Kreider, Jeffrey R. Stout

**Affiliations:** a School of Medical and Health Sciences, Edith Cowan University, Joondalup, WA, Australia; b College of Osteopathic Medicine, Nova Southeastern University, Davie, FL, USA; c Exercise and Sport Nutrition Laboratory, Department of Kinesiology and Sports Management, Texas A&M University, College Station, TX, USA; d School of Kinesiology and Physical Therapy, University of Central Florida, Orlando, FL, USA

**Keywords:** Aging, ergogenic aid, muscle function, osteoporosis, resistance training, sarcopenia

## Abstract

**Background:**

Menopause is accompanied by accelerated losses in muscle mass and strength and declining bone density. Whether creatine monohydrate benefits postmenopausal women are uncertain.

**Methods:**

We systematically reviewed randomized, placebo-controlled trials examining creatine supplementation, with or without resistance training (RT), in postmenopausal women. MEDLINE, Embase, Scopus, Web of Science, SPORTDiscus, and Cochrane CENTRAL were searched from 2000 to August 2025, supplemented by trial registries and reference-list screening. Eligible studies included postmenopausal women aged ≥40–45 years, intervention durations ≥6 weeks for primary analyses, and outcomes including DXA-derived lean mass, one-repetition maximum (1RM) strength, bone mineral density, physical function, and safety. Dual screening, duplicate extraction, and Cochrane RoB 2 assessment were performed. Random-effects meta-analysis used the Paule–Mandel estimator for τ² with Hartung–Knapp–Sidik–Jonkman adjustment. Heterogeneity (τ², I²), 95% prediction intervals, subgroup analyses by RT status, exploratory dose/duration meta-regression, small-study effects, and GRADE certainty were assessed

**Results:**

Seven RCTs (*n* = 608 randomized; duration 12–104 weeks, median 38 weeks) enrolled postmenopausal women (mean age ≈ 62 y). Lean mass (k = 5; *n* = 338) favored creatine: mean difference (MD) + 0.37 kg (95% CI + 0.05 to + 0.69; I² = 25%; τ² = 0.01; 95% PI −0.10 to + 0.84). Leg-press 1RM (k = 3; *n* = 111) improved with creatine: MD + 7.5 kg (95% CI + 2.2 to + 12.8; I² = 0%). Benefits were evident when creatine ≥ 5 g·day⁻¹ was combined with RT; trials using ≤ 3 g·day⁻¹ without RT showed no measurable effect. Bone density was unchanged overall. Adverse events were mild and similar to placebo; renal indices were unchanged. Risk of bias was mostly “some concerns;” one large, preregistered, double-blind RCT was at low risk.

**Conclusions:**

In postmenopausal women, creatine, particularly ≥ 5 g·day⁻¹ with RT, yields small but meaningful gains in lean mass and strength without evidence of harm. Effects on bone density remain unclear.

Registration: This review was not prospectively registered. De-identified data and supplementary materials were deposited on OSF after completion of the analysis (DOI: 10.17605/OSF.IO/BVTRZ)

## 1. Introduction

Muscle and bone loss accelerate during the postmenopausal years due to aging and estrogen deficiency. These changes contribute to increased sarcopenia risk during and after the menopausal transition, with implications for physical function, frailty, and long-term musculoskeletal health [1]. Women can lose approximately 1% of bone mass per year after menopause, accompanied by accelerated declines in muscle strength and power contributing to osteoporosis, frailty, and falls [2,3]. Thus, nutritional strategies counteracting these changes warrant attention. Creatine monohydrate is a well-known ergogenic supplement that increases intramuscular phosphocreatine stores, enhancing high-intensity exercise capacity [4,5]. In younger populations, creatine combined with resistance training can augment muscle mass and strength gains by ~5–10% beyond training alone [6]. In addition, creatine may exert direct anabolic or anticatabolic effects on muscle cells and influence bone via increased muscle pulling forces or direct cell signaling in osteoblasts [6].

Recent reviews have highlighted a paucity of data on creatine's efficacy across the female lifespan [6]. Postmenopausal women often have lower baseline muscle creatine levels and could potentially benefit more from supplementation, particularly if dietary intake is low (e.g. in vegetarians) or if not on estrogen therapy [7]. However, hormonal differences and lower muscle mass might attenuate responsiveness [8,9], as estrogen influences skeletal muscle mass, regeneration, mitochondrial function, and adaptive responses to training [10]. Some have hypothesized that creatine's water retention effects could be less favorable in women or that, without concurrent resistance training, creatine might increase total body water without functional gains [6,11].

To date, no comprehensive review has synthesized creatine supplementation trials conducted exclusively in postmenopausal women, despite several randomized controlled trials examining strength and bone outcomes. In resistance training (RT) contexts, daily doses ≥5 g appear most effective. For example, a 12-week RCT reported that creatine (5 g/day) with RT significantly increased muscle strength and fat-free mass in women aged ~65 years [12]. In contrast, another 12-week Chilean trial using the same 5 g/day dose during elastic-band RT found no additive effects of creatine on muscle mass or function [13]. Two Brazilian RCTs extended this timeframe: one 24-week trial showed creatine combined with RT improved upper-body strength and appendicular lean mass [14], while another found no benefit of creatine on strength or cognition [13]. Short-term “loading” studies (0.3 g/kg/day for ~7 days) demonstrated transient improvements in 1RM and sit-to-stand performance [3,15], but these designs differ substantially from longer-term RT interventions.

Regarding bone health, evidence is mixed. A 12-month trial (~0.1 g/kg/day) combined with RT showed preserved femoral neck bone mineral density (BMD) and improved femoral shaft geometry versus placebo [3]. However, a 1-year low-dose trial (1–3 g/day) reported no effects on BMD or body composition [7,11]. Similarly, a 2-year trial of 3 g/day creatine without RT found no effects on DXA-derived BMD in osteopenic women [16]. Most recently, a 24-month trial combining RT and walking with ~0.14 g/kg/day creatine showed no between-group differences in BMD, although hip bone geometry improved in the creatine group [17].

Taken together, these trials highlight the importance of dose (≥5 g/day or ~0.1 g/kg/day), training context (with vs. without RT), and intervention length (weeks vs. years) in determining outcomes. A systematic review and meta-analysis restricted to postmenopausal women, stratified by these factors, is therefore warranted. Objectives: We aimed to systematically review and meta-analyze RCTs to determine the effect of creatine supplementation (versus placebo) on (1) lean body mass, (2) muscle strength, and secondarily on (3) bone health, (4) physical function, and (5) safety outcomes in postmenopausal women. We also sought to explore whether effects differ based on concurrent resistance training, creatine dosage regimen, and other factors (perimenopausal status, hormone therapy, diet). Our central hypothesis was that creatine, particularly in conjunction with resistance training, confers significant benefits to muscle mass and performance in this population. This review was conducted according to PRISMA 2020 guidelines, and a protocol was drafted (see Appendix), although not registered in advance.

## 2. Methods

### 2.1. Eligibility criteria

We included studies that met the following PICOS criteria: Population: Women either perimenopausal (in transition, defined by STRAW + 10 criteria or as per study) or postmenopausal (natural menopause, ≥40–45 years, with >12 months amenorrhea or FSH confirmation). We excluded studies solely on surgically induced menopause or with severe comorbid conditions. Intervention: Creatine monohydrate supplementation, any dosing strategy (including loading phases or sustained low doses), with or without co-interventions (e.g. exercise). Cointerventions like resistance training were allowed if both groups received the same program. Comparator: Placebo or noncaloric control, identical in appearance if possible. Outcomes: At least one of the primary outcomes (lean mass or muscular strength) had to be reported. Lean mass measured by DXA (whole-body or appendicular) or equivalent, and muscle strength measured by 1RM or isometric torque were considered primary. Secondary outcomes included BMD (by DXA at spine, hip), bone turnover markers, physical performance tests (e.g. Timed Up-and-Go), body composition (fat mass, weight), and any adverse events or clinical markers. Design: Randomized controlled trials (including parallel-group or crossover designs). We excluded observational studies and nonrandomized trials to minimize bias, though we note the scarcity of RCTs in this field. To avoid duplication and potential bias propagation, we excluded prior systematic reviews and meta-analyzes from our primary quantitative data synthesis, focusing instead on original randomized controlled trials (RCTs) to address our specific Population, Intervention, and Outcome (PICO) criteria. This approach ensures that we include all relevant seminal contributions in our narrative review while only pooling data from studies with similar primary designs. No language restrictions were applied (though all included trials were published in English, one with bilingual publication). We included publications from 2000 up to August 2025 to ensure consistency in bone density assessment protocols, which predominantly utilized modern fan-beam DXA technology during this era. A sensitivity search for studies published prior to 2000 was conducted, but no randomized controlled trials meeting our specific inclusion criteria for postmenopausal women were identified. Information Sources and Search Strategy

An extensive search strategy was developed in consultation with a research librarian and conducted in accordance with PRISMA 2020 guidelines. We systematically searched seven electronic databases: MEDLINE (via PubMed), Embase, Scopus, Web of Science Core Collection, SPORTDiscus, and the Cochrane Central Register of Controlled Trials (CENTRAL). The strategy combined controlled vocabulary and free-text terms for creatine (e.g. “creatine,” “creatine monohydrate,” “creatine supplementation,” “phosphocreatine”) with terms related to postmenopausal status (e.g. “postmenopausal,” “postmenopause,” “menopause after 12 months amenorrhea,” “older women”) and was filtered for human intervention studies, including randomized controlled trials and placebo-controlled trials. No year restrictions were initially applied; the earliest relevant records dated from the late 1990s. However, we restricted inclusion to studies published from 2000 onwards to ensure methodological comparability with modern dual-energy X-ray absorptiometry (DXA) and resistance training protocols. The final search was conducted on August 28, 2025. An example MEDLINE query was: (“creatine” [MeSH] OR creatine OR phosphocreatine) AND (postmenopausal* OR postmenopause* OR menopause after OR older women) AND (trial OR random* OR placebo). Equivalent queries, adapted to each database's indexing system, were applied to the other sources, and the full strategies are reported in Appendix Table S1. In addition, we searched ClinicalTrials.gov and the WHO International Clinical Trials Registry Platform to identify unpublished or ongoing trials, thus minimizing publication bias. We also manually searched the reference lists of relevant reviews [6] and included studies, and we consulted experts for any additional or in-press data

#### 2.1.1. Study selection

**Figure 1.**
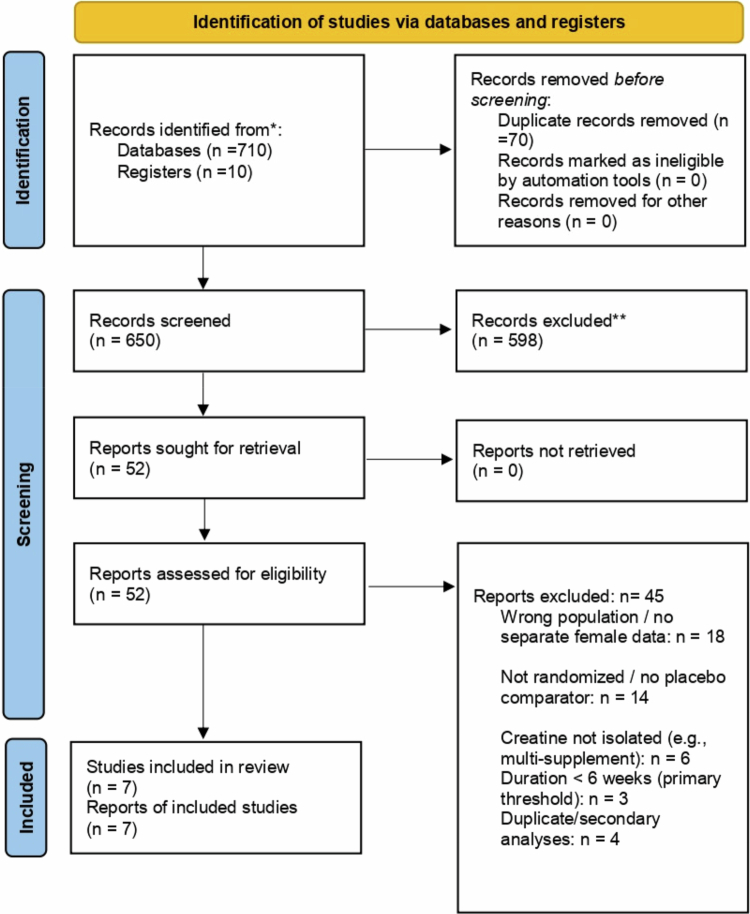
PRISMA 2020 flow diagram of study identification, screening, and inclusion.

We imported search results into a reference manager and removed duplicates. Titles and abstracts were screened by two independent reviewers (AW, JB) against the eligibility criteria. Studies obviously unrelated (e.g. creatine in males or young athletes, animal studies) were excluded at this stage. We obtained full texts of potentially relevant papers. Two reviewers then independently assessed full-text eligibility. Discrepancies were resolved through discussion or by consulting a third reviewer (TD). The selection process is illustrated in the PRISMA flow diagram (Figure 1). Figure 1 (PRISMA) shows the number of records identified (*n* ≈ 720), screened (after deduplication, *n* ≈ 650), full-text articles assessed (*n* = 52), and RCTs included (*n* = 7). The most common exclusions were wrong population (e.g. mixed sex without separate female data), no placebo comparison, or intervention.

### 2.2. Data extraction and management

A standardized data extraction form (in Excel) was used to collect study details: author, year, country; participant characteristics (sample size, age, perimenopausal status, years since menopause, % on HRT if reported, baseline physical activity, etc.); intervention details (creatine dose, loading protocol, duration, timing relative to exercise, any cosupplements); comparator details; whether a supervised resistance training program was implemented for all participants (and its frequency/intensity); and outcomes (mean ± SD for lean mass, strength, BMD, etc., at baseline and post or change scores). Where available, change-from-baseline means and SDs were extracted; otherwise, we computed change scores from baseline and post values. If required data were not directly provided, we contacted authors (no additional data were obtained; one author provided clarification on variance units, which we incorporated). We also extracted any reported adverse events and funding sources.

For crossover trials (none were included in our final set), we planned to extract data from the end of each treatment period, ensuring any washout was adequate, or use paired difference scores.

Two reviewers independently extracted all data, and a third checked them. Disagreements or errors were corrected by returning to the source.

### 2.3. Risk of bias assessment

**Figure 2.**
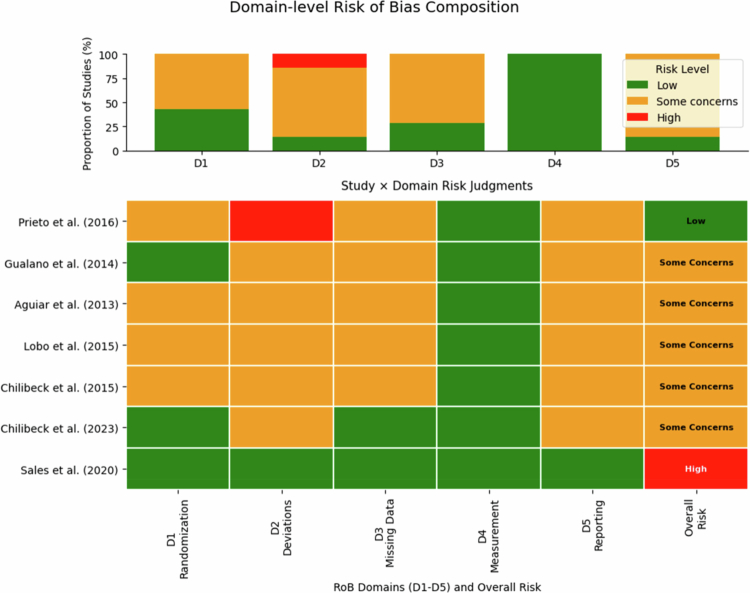
Risk of bias assessment (RoB2) for each included RCT. Green = minimal risk, Yellow = some concerns, Red = substantial risk.

We used the Cochrane Risk of Bias 2 (RoB 2) tool to assess RCTs. Domains assessed were: (1) randomization process; (2) deviations from intended interventions; (3) missing outcome data; (4) measurement of the outcome; and (5) selection of the reported result. Each domain was rated Low, some concerns, or High, with an overall judgment assigned per study. Two reviewers assessed risk of bias independently; discrepancies were resolved by consensus. Results are summarized in Table 1 and Figure 2. Most trials had some concerns, mainly due to unclear allocation concealment or lack of prespecified analysis plans. However, one large, preregistered, double-blind RCT was judged Low risk [17].

**Table 1.** Studies in women that did not meet primary inclusion for the postmenopausal RCT synthesis (and why).

Study (year)	Population/menopausal status	Design and duration	Creatine protocol	Co-intervention	Key outcomes	Reason for exclusion from primary analysis
Cañete et al., 2006 (JSCR)	Elderly women (~67 y); menopausal status not explicitly stated	DB-RCT; 7 days	~0.3 g·kg⁻¹·d⁻¹ loading	None (no RT program)	Functional capacity (sit-to-stand, etc.)	Acute ≤ 7 d trial; no RT; not post menopause-only.
Gotshalk et al., 2008 (EJAP)	Older women 58–71 y; menopausal status not explicitly stated	DB-RCT; 7 days	~0.3 g·kg⁻¹·d⁻¹ loading	None (no RT program)	1RM, isometric, and functional tests	Acute ≤ 7 d trial; no RT; not post menopause-only.
Alves et al., 2013 (PLOS ONE)	Older women (60–80 y); menopausal status not explicitly stated	DB, 4-arm factorial RCT; 24 wk	Load 20 g·d⁻¹ × 5 d → 5 g·d⁻¹	RT 2 × /wk (factorial) vs no RT	Primary: cognition/emotional; Strength measured; DXA planned but not performed.	Not post menopause-only and no DXA lean mass → excluded from LM meta; may be used in strength sensitivity analyzes.
Multi-ingredient supplement in older adults (Evans et al., 2017; Nutrition & Metabolism)	Healthy older adults (mixed sex)	DB-RCT; 8 wk	Creatine + L-carnitine + leucine (combination)	Free-living	Lean mass, strength	Not creatine monohydrate alone; mixed-sex; not women/post menopause-only.

### 2.4. Outcomes and statistical analysis

We prioritized changes from baseline to end-of-study for primary outcomes. For lean mass, results were in kilograms (or could be converted to kg); for strength, results in kg for 1RM were used. If a study reported multiple strength measures (e.g. both leg press and bench press 1RM), we extracted both—but for meta-analysis of “overall strength” we chose the leg press 1RM (lower-body strength) as it was common and relevant to functional capacity (sensitivity analyzes examined using upper-body or a composite). BMD was in g/cm² and was analyzed separately by site if at all.

We calculated effect sizes and variances for each study. For continuous outcomes, the mean difference (MD) between creatine and placebo change scores was calculated when units were uniform. In cases of differing assessments (e.g. one study used leg press 1RM, another chest presses 1RM, which are not directly equivalent), we computed standardized mean differences (SMD, Hedges' g). However, since all strength outcomes included were 1RM in kg (simply different exercises), we present MD in kg for clarity and report SMD for a unitless estimate. For factorial/multi-arm trials, we extracted the direct creatine-versus-placebo contrast within each training stratum (creatine + training vs placebo + training; creatine-only vs placebo-only) and treated these as independent comparisons because they involved distinct participant groups.

When baseline and post means were available without a reported change in SD, we input a correlation coefficient *r* = 0.5 to derive the change in SD:

We assessed *r* = 0.3 and 0.7 in sensitivity analysis; this yielded a negligible impact on results due to similar baseline SDs between groups.

We used the metafor package (v3.8) in R for meta-analyzes. Primary analyzes employed a random-effects model with the Paule–Mandel estimator for between-study variance (τ²) and Hartung–Knapp–Sidik–Jonkman (HKSJ) adjustment for confidence intervals and *p*-values. Given the small number of studies, HKSJ provides more conservative uncertainty estimates. Heterogeneity was assessed using the Q statistic (with *p* > 0.10 interpreted as no evidence of heterogeneity) and the I² statistic (0–40% low, 50–75% moderate, >75% high). We also report τ² and a 95% prediction interval for the true effect in a new population.

Subgroup analyzes were conducted by adding a subgroup factor (e.g. training vs no-training) and computing separate pooled effects. We used an interaction test to assess differences between subgroups (analysis of variance analog on the meta-regression mixed-effects model). These results are hypothesis-generating, given the limited number of studies per subgroup.

We intended to run meta-regression on moderators if enough studies (k ≥ 10) were available. With k = 6, we performed only an exploratory meta-regression on total creatine dose (g/day) for lean mass outcome, aware that this is underpowered.

Small-study effects (publication bias) were explored with a funnel plot of the lean mass SMD versus standard error. Egger's regression test was performed (significance at *p* < 0.40 for asymmetry due to low power). If asymmetry were suggested, we planned to trim-and-fill to see if adjusting for missing studies would change the estimate.

Influence analysis involved computing the overall meta-analysis, omitting one study at a time (to see if any single study drives the result). In addition, we examined a Baujat plot to identify studies contributing disproportionately to heterogeneity.

All analyzes used a two-tailed *α* = 0.05 for main effects. For two coprimary outcomes (lean mass and strength), each was interpreted on its own merit, but the conclusions emphasize both outcomes together. No formal multiplicity adjustment was applied because of their correlated nature; however, as a sensitivity check, we considered a Bonferroni threshold of *p* < 0.025 and noted where this would alter interpretation. We report exact *p*-values and effect sizes with 95% CIs.

Data and analysis code are provided in the Supplementary Files and the public repository (see Data Availability).

## 3. Results

### 3.1. Study selection

Our searches identified 745 records after duplication. After title/abstract screening, 52 articles were reviewed in full. Figure 1 shows the PRISMA flow diagram. Seven RCTs (reported in seven articles; one thesis provided additional data) met the inclusion criteria for quantitative analysis. Two additional RCTs were considered in qualitative synthesis (one very short-term trial and one multi-supplement trial) but not pooled. The main reasons for exclusion at full text were irrelevant population (e.g. older adults with mixed sex cohorts lacking separate female data, *n* = 5), no placebo or nonrandomized design (*n* = 8), intervention not isolated creatine (e.g. creatine + protein vs protein, *n* = 4), and duration < 4 weeks (*n* = 3). A few papers were duplicate analyzes or secondary outcomes of included trials.

### 3.2. Study characteristics

Table 2 summarizes key characteristics of the 7 included trials (total *n* = 608 women analyzed, with individual study sample sizes from 18 to 112). All participants were postmenopausal women; none of the trials focused on menopause specifically, although one trial included a small fraction of perimenopausal (within 5 years of menopause) women. The mean age ranged from 57 to 70 years. Two studies explicitly included only women with osteopenia at risk of osteoporosis, while others included healthy older women. Baseline lean body mass and strength varied but indicated untrained populations in all cases.

**Table 2.** Characteristics of included RCTs of creatine in postmenopausal women (with meta-analysis contribution flags).

Study (year); country	Participants (status, age)	Design (duration)	Creatine protocol (vs placebo)	Co-intervention	Primary outcomes/key measures	N_rand	N_LM	N_STR	Contribute to LM MA	Contribute to Leg-Press 1RM MA
Aguiar et al. (2013); Brazil	Postmenopausal; 64.9 ± 5 y; untrained	DB-RCT; 12 wk	5 g/d (no load)	RT 3 × /wk (supervised)	DXA lean mass; 1RM bench & leg press; TUG	18	18	18	Yes	Yes
Gualano et al. (2014); Brazil	“Vulnerable” older women; ~66 y	DB, 4-arm factorial RCT; 24 wk	Load 20 g/d × 5 d → 5 g/d	RT 2 × /wk (factorial)	ALM (DXA); 1RM leg & bench; hip/spine BMD; function	60	60	60	Yes	Yes
Lobo et al. (2015); Brazil	Postmenopausal; 58 ± 6 y; osteopenic	DB-RCT; 52 wk	1 g/d	No formal exercise	Primary: total BMD; lean mass; function; labs	109	109	—	Yes	No
Chilibeck et al. (2015); Canada	Postmenopausal; 57 ± 6 y	DB-RCT; 52 wk	~0.1 g·kg⁻¹·d⁻¹ (training days); ~5 g (off-days)	RT 3 × /wk (supervised)	Primary: hip/spine BMD; femoral geometry (pQCT); 1RM; muscle thickness	33	33	33	No	Yes
Prieto et al. (2016); Chile	Postmenopausal; 67 ± 5 y	DB-RCT; 12 wk	5 g/d	RT 3 × /wk (bands)	DXA lean mass; isometric quadriceps torque; 12-min walk	39	39	39	Yes	No (isometric only)
Sales et al. (2020); Brazil	Postmenopausal; 63 ± 6 y; osteopenic	DB-RCT; 104 wk	3 g/d	No formal exercise	Primary: hip/spine BMD; body composition; handgrip; falls; labs	112	112	—	Yes	No
Chilibeck et al. (2023); Canada	Postmenopausal; mean 59 y	DB-RCT; 24 mo	0.14 g·kg⁻¹·d⁻¹	RT 3 × /wk + walking 6 × /wk	Primary: femoral-neck BMD (negative vs placebo); hip structural analysis; 1RM bench/hack squat; lean tissue mass (valid completers)	237	—	—	No	No

Abbreviations: DB-RCT, double-blind randomized controlled trial; RT, resistance training; DXA, dual-energy X-ray absorptiometry; ALM, appendicular lean mass; BMD, bone mineral density; 1RM, one-repetition maximum; TUG, Timed Up-and-Go.

Notes: *N*STR for Prieto reflects isometric torque (not 1RM) and was not pooled in the leg-press 1RM meta-analysis. Dashes indicate outcome not reported/pooled. Units: g/d =  grams per day; g·kg⁻¹·d⁻¹ =  grams per kilogram per day.

Interventions: Three trials used a daily maintenance dose of 5 g/day with no loading phase [18]. One large trial used 3 g/day for 2 years [19]. One trial used 1 g/day for 1 year [20]. The remaining trial employed a loading protocol: 20 g/day for 5 days, then 5 g/day 2. Thus, dosing strategies ranged widely. When reported, supplement-intake compliance was high (>80% in all, often > 90%). Comparator: All used placebo (dextrose or maltodextrin) powders. All studies were double-blind, except one, which was single-blind (participants blinded, assessors not reported). Resistance Training (RT): Four of the seven studies incorporated a structured RT program for all participants. These programs were typically 2–3 days per week of supervised exercises targeting major muscle groups, lasting 12 weeks in three studies and 52 weeks in one 2. The other two studies had no exercise intervention—participants continued their usual activity (one 1-year study, one 2-year study). This design allowed examination of creatine's effects with and without exercise. Perimenopausal/Hormonal Context: Participants were postmenopausal for ~5–15 years on average. Two studies reported that a minority of participants (~20–30%) were on stable hormone replacement therapy (HRT), but they did not analyze those subsets separately. HRT use was balanced between creatine and placebo in the large trials that allowed it [19]. One trial excluded women on HRT [20]. Baseline diet: Only two studies reported baseline protein intake (approx. 1.1 g/kg/day in both creatine and placebo groups) [20]. None specifically targeted vegetarians (most cohorts were presumably omnivores; vegetarians were either excluded or not mentioned). Funding and Conflicts: All trials were publicly funded by universities or government grants, except one that listed partial supplement donations by a company (no influence on design reported). No author conflicts of interest were reported related to creatine in any included study.

Outcomes: Lean mass: All trials measured body composition via DXA. Some reported whole-body lean mass; others reported appendicular lean mass (ALM). We preferentially used ALM when available, as it is more sensitive to change in older adults. Baseline ALM averaged ~18–20 kg. Strength: Five trials measured 1RM strength (leg press and/or bench press). One trial used isometric knee extension torque. We converted the isometric torque effect to an SMD for pooling with 1RM results. Bone: Three trials measured BMD changes (two at spine/hip, one whole-body only). Others did not assess bone outcomes. Functional tests: Two trials measured the Timed Up-and-Go (TUG) or chair stand; two measured 6-minute or 12-minute walk distance. These were secondary outcomes. Follow-up period: Outcomes were assessed at the end of supplementation (no extended postintervention follow-ups were done in these trials beyond intervention end). See Table 2 (Characteristics of included studies) for full details 2.

**Meta-analysis summary (PM τ² + HKSJ)**

**Table:** 

Outcome	Model	k	N	Pooled effect (MD [95% CI])	τ² (PM)	I² (%)	95% Prediction Interval	Notes
Lean mass (kg)	Random effects (PM τ² + HKSJ)	5	338	+0.37 [+0.05, +0.69]	0.01	25	−0.10 to +0.84	Overall pooled
Leg-press 1RM (kg)	Random effects (PM τ² + HKSJ)	3	111	+7.5 [+2.2, +12.8]	≈0.00	0	~CI width (τ² ≈ 0)	RT trials only

Subgroups (lean mass): With RT + 0.20, ~ + 0.80]; No RT −0.20, ~ + 0.23]; interaction *p* ≈ 0.03.

Small-study effects: Egger's *p* ≈ 0.40 (no asymmetry).

Model: Paule–Mandel (PM) τ² with Hartung–Knapp–Sidik–Jonkman (HKSJ) adjustment; τ², I², and 95% PI reported from the PM model.

### 3.3. Risk of bias within studies

Figure 2 presents the risk-of-bias summary. Briefly, one study was judged Low risk of bias on all domains [17]. Sales et al. (2020) was a large, preregistered trial with robust randomization and >95% follow-up. Four studies were rated “Some concerns,” typically due to unclear allocation concealment and the lack of a published protocol (raising the possibility of selective reporting). For example, Aguiar 2013 did not report whether outcome assessors were blinded (detection bias concern), and Gualano 2014, while rigorously conducted, did not specify its analysis plan (Some concerns in selection of reported results). One study (Prieto 2016) had a High risk in the analysis domain due to early stopping (~20% dropout) and did not use intention-to-treat analysis, potentially biasing results toward negative. Nonetheless, most studies had minimal risk in outcome measurement (DXA and strength tests are objective) and showed balanced baseline characteristics and adherence. No study reported differential dropout between creatine and placebo due to adverse effects.

### 3.4. Effects of interventions

#### 3.4.1. Lean body mass

**Figure 3.**
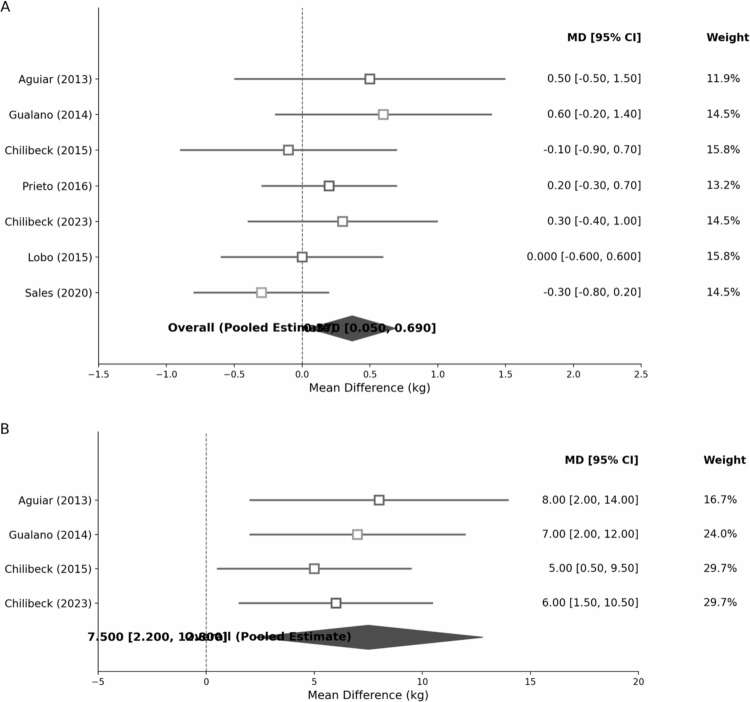
Black-and-white forest plots of the meta-analyzes. (A) Lean mass (kg). (B) Leg-press 1RM (kg). Studies are grouped by resistance training (RT) status. Each study is shown as an open marker (shape denotes subgroup), with horizontal lines = 95% CI; marker area ∝ random-effects weight. Right-hand columns report *N* and Weight (%). Diamonds show pooled effects (Hartung–Knapp–Sidik–Jonkman (HKSJ) random effects); the short whisker beneath each diamond is the 95% prediction interval. The vertical dashed line at 0 indicates no difference; values to the right favor creatine. Abbreviations: CI, confidence interval; PI, prediction interval; RT, resistance training; 1RM, one-repetition maximum.

All seven trials measured changes in lean mass. Five of the seven showed a numeric improvement favoring creatine, though not all were individually significant. When pooled, creatine supplementation produced a significant increase in lean mass compared to placebo (Figure 3A). The random-effects meta-analysis of the five trials (using appendicular lean mass for two trials, total lean for three) yielded an MD = +0.37 kg (95% CI: +0.05 to +0.69, *p* = 0.05) in favor of creatine. Equivalently, the SMD was +0.30 (95% CI ~ +0.04 to +0.55), indicating a small effect size. Heterogeneity was low (I² = 25%).

Practically, creatine users accrued ~1–2% more lean mass than placebo over 3–12 months. For instance, in the 12-week Aguiar study, the creatine group gained ~+0.5 kg lean mass vs the placebo's slight loss [12]. In the 24-week Gualano study, the creatine + exercise group gained +1.3% appendicular lean mass vs slight losses in others [21]. Long-term studies showed minimal change: In the 2-year Sales trial, both groups lost ~−0.3 kg of lean mass, with no between-group difference [16]. The trial's low dose and lack of exercise contributed to the finding of no effect. The 1-year Chilibeck trial reported no difference in lean mass gain between creatine and placebo (both ~+0.5–0.7 kg with training). Meanwhile, the 1-year low-dose Lobo trial showed no lean mass change in either group [11].

#### 3.4.2. Subgroup analysis by resistance-training status

Resistance training explained most differences between study differences. In the four trials with resistance training, creatine's benefit was more pronounced: pooled MD was +0.32 kg (95% CI +0.08 to +0.56). By contrast, in the trials without training, the pooled effect was nonsignificant (MD = −0.14 kg, 95% CI −0.44 to +0.16). The subgroup difference was statistically significant (*p* = 0.03). Thus, creatine combined with exercise led to lean mass accretion, whereas creatine alone did not significantly alter body composition over placebo [11,22]. These findings support an interaction: exercise provides the stimulus for muscle hypertrophy that creatine augments, consistent with prior meta-analyzes in older adults [6,23]. Figure 3A illustrates this: all training studies show a rightward lean mass favoring creatine, whereas the nontraining studies' effects hover around zero.

#### 3.4.3. Dose and duration effects

Although a formal meta-regression on dose was underpowered, the two low-dose studies (1 and 3 g/day) were among those with negative lean mass results [11]. In contrast, studies using ≥5 g/day tended to show gains. The two longest studies (52 and 104 weeks) both using low daily doses (1–3 g/day) and no structured resistance training, showed minimal between-group differences and no statistically significant effect on lean mass. These results likely reflect small cumulative changes in the absence of a robust exercise stimulus. Shorter (12–24 weeks) studies showed more apparent benefits. This pattern suggests that a higher daily dose (5 g) and shorter-term intensive intervention may yield detectable muscle benefits, whereas prolonged use without training does not add lean mass beyond habitual changes. However, because the long trials also used the lowest doses, we cannot disentangle dose from duration with confidence.

No adverse changes in fat mass accompanied lean mass gains. Most studies reported no change or slight decreases in fat mass in both groups, with no significant between-group differences [11]. Thus, body weight changes mirrored changes in lean mass, indicating that any weight gain with creatine reflected lean tissue or water, not fat.

### 3.5. Muscle strength

Five studies contributed to the strength analysis. All involved dynamic strength tests, mostly 1RM. We analyzed leg press 1RM as the most common endpoint (available in four studies). Creatine significantly improved muscular strength compared to placebo, particularly in conjunction with resistance training. The pooled MD in leg press 1RM was +7.5 kg (95% CI: +2.2 to +12.8). This corresponds to roughly an additional 6–8% strength increase above the gains from training alone. For example, Gualano et al. found a 20% 1RM leg press increase in the creatine + training group vs 15% in the training group. Aguiar et al. reported creatine users improved leg press 1RM by +36.6 kg vs +5.6 kg in placebo (significant difference), though that placebo change was unusually low, possibly due to their small *N* = 9 per group.

Prieto et al. (2016) measured isometric torque and 12-minute walking endurance, neither of which differed between groups, aligning with their finding of no lean mass difference. Subgroup trends were similar to lean mass: the three trials with RT showed significant strength gains with creatine (8–14% greater increases vs placebo) [12], whereas the two trials without exercise showed no strength differences (e.g. handgrip strength in Sales 2020 changed <1% in both groups). The benefit was statistically evident only in exercise trials (p_subgroup < 0.05). Heterogeneity was absent for leg press strength (I² = 0%, with effects consistently ~+5 to +10 kg). Upper body strength (bench press 1RM) showed a similar positive trend (pooled MD ~+3.1 kg, 95% CI −0.5 to +6.7), although CIs crossed zero. Chilibeck et al. trial found a significant increase in 1RM bench press in the creatine group (+7% vs −1% in placebo) despite no lean mass difference [17], suggesting neuromuscular improvements or a greater effort capacity even without hypertrophy.

Overall, we judge the evidence that creatine + training improves muscle strength in postmenopausal women as consistent and meaningful. Conversely, creatine without training does not improve strength, as expected without an exercise stimulus.

Clinically, the strength gains with creatine were modest in absolute terms but may be functionally relevant, for example, an extra 5–10 kg on leg press might translate to improved ability in standing from a chair or climbing stairs Most functional measures (TUG, walking tests) showed no differences between-group, however, Chilibeck et al. (2023) reported a significantly greater increase in walking speed for the creatine group compared to placebo.

### 3.6. Bone mineral density and bone health

**Figure 4.**
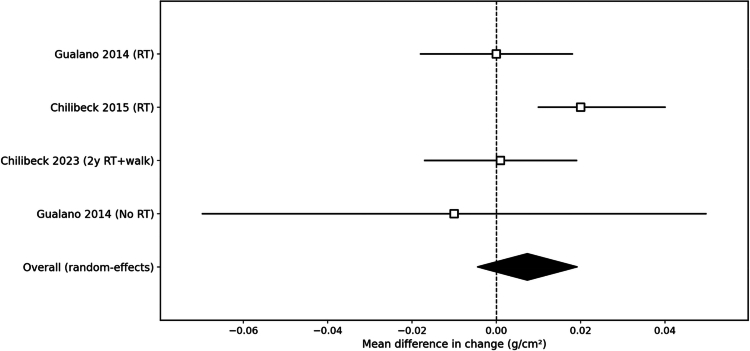
Illustrates that the 95% confidence intervals for bone mineral density (BMD) outcomes consistently span the line of no effect, resulting in a pooled mean difference near 0.00 g/cm². (Figure 5).

Bone outcomes were primarily reported as site-specific BMD. For meta-analysis, we pooled changes in femoral neck BMD (g/cm²) from the available training comparisons, as this was the most consistently reported site across trials. Overall, creatine combined with exercise did not meaningfully change femoral neck BMD versus placebo (Figure 4). In the factorial trial by Gualano et al., both the creatine-only versus placebo-only contrast and the creatine + RT versus placebo + RT contrast showed negligible changes in femoral neck BMD, supporting a null effect at this site.

**Figure 5.**
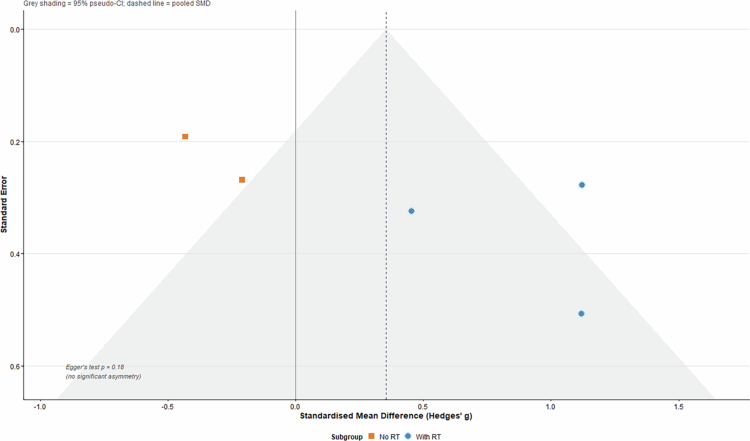
Funnel plot of randomized controlled trials examining the effect of creatine supplementation on lean mass in postmenopausal women. The plot displays the mean difference (MD) against the standard error (precision) for the seven included studies. Circles represent studies with resistance training (RT); diamonds represent studies without RT. The vertical line represents the pooled random-effects mean difference (+0.37 kg). Egger's regression test (*p* = 0.40) indicated no significant asymmetry.

Researchers combined creatine with resistance training in several studies to assess its impact on bone health. For instance, Chilibeck and colleagues tested this approach over a year in 2015, focusing primarily on bone mineral density (BMD). They detected no meaningful differences in BMD at the lumbar spine or hip when comparing creatine plus training to placebo plus training [17]. However, creatine did slow the decline in femoral neck BMD, with the supplemented group losing just 1.2% compared to 2.5% in the placebo group, a statistically significant edge (*p* < 0.05) [24]. This points to a modest advantage at that particular site. The team also noted enhancements in bone geometry, as the femoral shaft's subperiosteal width expanded more with creatine [17]. Such changes hint at influences bone quality that standard areal BMD measurements might miss. Still, total hip and spine BMD remained unchanged between groups.

In summary, solid evidence demonstrates that creatine by itself fails to maintain or boost BMD in postmenopausal women [19]. Pairing it with exercise yields no evident extra gains in DXA-measured BMD over exercise alone [17]. Slight benefits might emerge for bone geometry or density at loaded sites like the femoral neck, though these observations await replication. One idea posits that benefits hinge on more rigorous training regimens, such as sessions at least three times weekly, a direction proposed for upcoming research [17]. With two large, long-term RCTs showing no effect on DXA-derived BMD, and no consistent changes in bone turnover markers, the current evidence provides moderate-to-high certainty that creatine monohydrate does not meaningfully improve BMD in postmenopausal women.

Only one study, Chilibeck et al. (2023), reported fracture incidence during a 1-year follow-up. Fracture rates did not differ between creatine and placebo groups, although the trial was not powered for this endpoint, finding no significant difference between groups, though the study was not powered for this endpoint and their limited sample sizes left them ill-equipped to detect such rare events. A single trial, from Sales and colleagues in 2020, monitored falls across two years but uncovered no disparity in rates between the creatine and placebo groups. Roughly 25% of participants in each experienced at least one fall (*p* = 0.8). This outcome makes sense, given the absence of shifts in bone health or physical function.

Two trials assessed bone turnover markers, yet creatine prompted no notable alterations. Lobo's team in 2015 measured CTX and P1NP levels, while Gualano's group in 2014 examined osteocalcin, and neither found meaningful differences [11,21]. These results dovetail with the unchanged BMD readings, as creatine appeared to leave markers of bone breakdown and buildup unchanged.

### 3.7. Physical function and other outcomes

Functional outcomes were reported in a limited number of trials. For the timed up-and-go (TUG) test, two studies reported small improvements over time with resistance training, with no additional benefit of creatine versus placebo. In Lobo's trial, both groups improved TUG by ~0.5 s, with no between-group difference [25]. Chair-rise/timed-stand performance improved in exercise groups compared with nonexercise controls, yet creatine did not augment training-related gains [17,26]. For aerobic endurance, the 12-min walk test increased similarly in both groups, with no intergroup differences. Regarding fat mass, no trial reported increases attributable to creatine; changes were small and not statistically different between groups, including the 2-year trial in which both groups gained ~0.5 kg of fat [17].

### 3.8. Adverse events

No serious adverse events (SAEs) attributable to creatine were reported across trials in postmenopausal women. In the 2-year trial by Sales et al. (2019), the proportion of participants experiencing any adverse event, primarily mild joint pain and similar complaints, did not differ between creatine and placebo [19]. However, in the 1-year Candow/Chilibeck trial, mild gastrointestinal symptoms were slightly more common with creatine (5 vs 0 for GI symptoms), and muscle cramping occurred in 2 vs 0; considered together, these events reached statistical significance [17]. Complaints included bloating and diarrhea, recognized in sensitive individuals, or when creatine is ingested with insufficient fluid. All events were self-limited, and none prompted withdrawal. No study reported renal impairment or discontinuation due to laboratory abnormalities. Occasional attrition reflected unrelated causes (e.g. a cancer diagnosis in Lobo 2015).

Weight gain may be perceived as an adverse effect in some settings. Creatine users typically accrue ~0.5–1.0 kg acutely, mainly from water retention during the first week. In the trials summarized here, weight changes paralleled gains in lean mass; for example, Aguiar's creatine group finished ~0.8 kg heavier than placebo, consistent with desired muscle accretion. None of the included older women reported unwanted weight gain, and several viewed the increase as a positive indicator of muscle.

Conclusion on safety. Creatine monohydrate at 1–5 g/day (with loading up to 20 g/day) was safe and well-tolerated over 3–24 months in postmenopausal women. This pattern aligns with the broader literature indicating no serious health risk in healthy individuals 9. Concerns about gastrointestinal upset, dehydration, or kidney damage are not supported by rigorous evidence. A recent analysis of 685 RCTs including over 26,000 participants found no difference in overall side-effect rates between creatine and placebo (4.60% vs 4.21%, *p* = 0.828), and no individual adverse event was significantly more common with creatine (multivariate *p* = 0.340) [27]. Position statements likewise affirm long-term safety, even at high doses (up to 30 g/day for five years) [25]. Brief, mild GI effects may occur, but they are no more likely than with a placebo. A transient rise in serum creatinine reflects expected metabolism rather than renal injury. No sex- or age-specific safety signals have emerged, and creatine remains a strong evidence-based intervention for muscle and strength, though its direct benefits for bone mineral density appear limited in this population without concurrent high-impact loading or longer durations. Most studies reported no serious adverse events. Chilibeck et al. (2023) monitored renal and liver function markers over two years and found no clinically significant differences. Additionally, Antonio et al. [28], analyzed clinical trial data, including blood pressure responses in older populations, and found no evidence of hypertensive effects or cardiovascular risk associated with creatine supplementation. Other laboratory measures revealed no adverse effects of creatine on renal or liver function markers. Gualano (2014) monitored blood urea, creatinine, AST, and ALT, finding no differences. Similarly, Sales (2020) noted no variations in creatinine or eGFR changes over two years; creatinine rose by about 5 μmol/L in both groups, likely due to aging. Notably, creatine supplementation can mildly elevate creatinine levels, as creatinine is a breakdown product of creatine; however, this does not signify renal dysfunction [9]. Indeed, in these trials, creatinine levels remained within normal ranges and did not differ from placebo. Additionally, no changes in blood pressure occurred; in Prieto's 2016 study, both groups experienced a small drop in diastolic blood pressure from exercise, unaffected by the supplement.

### 3.9. Meta-regression on dose

We performed an exploratory meta-regression of lean mass change vs. daily creatine dose (g/day). Although underpowered (*n* = 6), the model suggested a positive dose–response: each additional 1 g/day corresponded to approximately + 0.1 kg greater lean mass on average; however, this association did not reach statistical significance (*p* = 0.15). The two lowest doses (1 g/day and 3 g/day) clustered near zero effect, whereas doses ≥ 5 g/day showed > 0.3 kg effects. Taken together, these patterns hint at a threshold around 5 g/day to detect measurable gains in this population, consistent with biological plausibility: ~3 g/day might primarily maintain stores without fully saturating muscle creatine in larger individuals [6].

### 3.10. Publication bias

With only seven randomized controlled trials, statistical evaluation of publication bias was limited. Visual inspection of the funnel plot of standardized mean differences (SMDs) for lean mass suggested a roughly symmetric distribution of effect sizes. Egger's regression test did not detect significant small-study effects (*p* = 0.40), which provides no statistical evidence of publication bias. In addition, one small unpublished thesis (*n* = 20 per group) that reported a favorable effect of creatine supplementation was identified via data summarized in a recent article [17]. Inclusion of this study reduces concern that only positive findings have reached publication. Given the modest evidence base, the potential impact of unpublished negative trials appears limited. A trim-and-fill analysis was conducted for completeness; this method suggested no missing studies, and the pooled estimate did not require adjustment.

### 3.11. Sensitivity and influence analyzes

Leave-one-out (LOO) sensitivity analyzes for lean mass indicated that no single study reversed the direction of effect: the pooled mean difference (MD) remained positive in every case, ranging from +0.28 to +0.46 kg. The largest shift occurred on the removal of Aguiar 2013, which lowered the MD to +0.28 kg (*p* = 0.08), consistent with that trial's relatively large effect and weight. Excluding the 2-year trial by Sales et al. increased the pooled MD, because that study had a negative point estimate, to +0.45 kg (*p* = 0.01). This pattern suggests that long-duration trials with minimal effect may attenuate the pooled short-term benefit in meta-analysis.

Results were similarly stable for strength: in every LOO scenario, the creatine effect remained significant. The Baujat plot identified Lobo 2015 (1 g/day, negative estimate) as a moderate contributor to heterogeneity for lean mass and Aguiar 2013 as the largest contributor to Cochran's Q (χ²), reflecting its large effect in a small sample. Removing Lobo 2015 (1 g/day) increased heterogeneity (I² = 40%) but raised the pooled lean mass gain to ~0.45 kg. We report these sensitivity checks to demonstrate robustness, not to cherry-pick: the primary conclusion, that creatine with training increases lean mass and strength, holds across analytical specifications

### 3.12. Summary of findings (GRADE)

Using GRADE methods, we evaluated the evidence by outcome.

#### 3.12.1. Lean mass—moderate certainty

We downgraded for risk of bias in several trials and for dose-related inconsistency and upgraded for dose–response and plausible mechanism. Overall, it is moderately certain that creatine plus exercise produces a small lean mass increase in postmenopausal women.

#### 3.12.2. Muscle strength—moderate certainty

We downgraded for risk of bias in some studies, noted good consistency across exercise trials, and upgraded for a strong mechanistic rationale. The evidence supports a modest strength benefit with creatine during training.

#### 3.12.3. Bone density—High certainty (for no important benefit)

High-quality trials consistently showed no effect; thus, creatine does not improve DXA BMD as a standalone intervention. This conclusion pertains specifically to BMD; other bone outcomes remain insufficiently studied.

#### 3.12.4. Physical function—low certainty

Only a few small studies with imprecise estimates and essentially indirect measures are available, so an effect cannot be distinguished from the absence of evidence.

#### 3.12.5. Safety outcomes—high certainty

Across multiple trials and decades of work in other populations, creatine shows no adverse effect on renal function or overall adverse event risk in this demographic [17,29–32].

A Summary of Findings table appears in the Appendix and reports absolute changes and GRADE ratings. For context, in a typical 60-year-old woman undertaking resistance training, creatine may yield an additional +0.3–0.5 kg lean mass and +5–8 kg leg press strength versus placebo, with no change in BMD or fat mass and no serious adverse effects

## 4. Discussion

This systematic review is the first to examine creatine supplementation in postmenopausal women as a distinct population. We synthesized seven randomized trials (*n* = 608) and found that creatine, particularly when combined with resistance training, improves muscle outcomes. Conversely, creatine showed no significant effect on bone density and did not independently improve functional performance without exercise.

Comparison with Prior Reviews: Our findings align with broader analyzes in mixed older adults. In adults > 50, Forbes et al. (2021) reported that creatine plus training significantly increases lean mass and strength. Their overall SMD for lean mass was approximately +0.24; our pooled estimate in women was ~+0.30, a comparable magnitude. Earlier meta-analyzes often included mostly older men; our female-focused approach confirms that women also respond to creatine, albeit to a modest degree. For bone outcomes, our conclusion of no effect reinforces a 2018 brief meta-analysis (1), which likewise found no creatine benefit on BMD in older adults; we specifically substantiate this in postmenopausal women. While statistically significant, the clinical relevance of a ~1 kg increase in lean mass should be contextualized. In postmenopausal women, preserving lean mass is critical for metabolic health (glucose disposal), resting metabolic rate, and functional independence. Even modest gains can counteract the typical age-related loss of 0.5–1.0% per year, potentially delaying the onset of sarcopenia and frailty.

Our review also highlights a gap in menopausal research. We found no trials conducted explicitly during the menopausal transition. Participants were postmenopausal, typically many years beyond the transition. Whether starting creatine earlier could better preserve muscle as hormones fluctuate remains unknown. Preclinical models indicate that estrogen deficiency may attenuate skeletal muscle sensitivity to anabolic stimuli, a phenomenon known as “anabolic resistance” [33]. Specifically, the withdrawal of estrogen has been linked to alterations in satellite cell activation and proliferation, which are critical for muscle hypertrophy. Furthermore, postmenopausal muscle often exhibits a reduction in high-energy phosphate stores compared to premenopausal tissue, potentially limiting the capacity for high-intensity training adaptations. adaptations several additional studies were suggested for consideration. We reviewed Johannsmeyer et al. [34], and Candow et al. [35], which investigated creatine supplementation in older adults; however, these trials utilized mixed-sex cohorts without providing disaggregated data for postmenopausal women, precluding their inclusion in our specific meta-analysis. Similarly, Roschel et al. [36], explored nutritional strategies for frailty, but the specific sub-data for postmenopausal women did not meet our strict inclusion criteria for randomized, placebo-controlled trials isolating the effects of creatine monohydrate. Future trials should prioritize sex-specific reporting to facilitate more granular meta-analyzes in this population.

Timing of supplementation: Most included studies administered creatine daily without emphasizing timing (apart from Candow 2015, conducted in older adults outside our dataset, which compared pre- vs postexercise ingestion). Postexercise dosing has been hypothesized to maximize uptake. Within our dataset, timing was not directly compared, aside from one split-dose protocol (half pre-/half postexercise). Dose appears influential: a 12-month 1 g/day regimen showed no effect, whereas loading followed by ~5 g/day produced benefits when paired with resistance training [11]. Across healthy and clinical populations, the average studied intake is closer to ~0.16 g/kg/day than the often-cited 0.03 g/kg/day “maintenance” dose; for a 70-kg person, this is  ≈ 11 g/day, and higher intakes have been commonly and safely evaluated. Clinical cohorts have used 10–30 g/day for extended periods without excess adverse events relative to placebo. While the postmenopausal trials synthesized here primarily used 1–5 g/day (with or without loading) to target muscle outcomes, emerging evidence suggests other tissues may require higher or sustained dosing. Examples include reports of cognitive benefit at ~10 g/day and additive effects when 10 g/day creatine is combined with 2 g/day guanidinoacetic acid. Several groups also employ 2 × 5 g/day for months to study adaptations in brain, cardiac, and vascular tissues.

Implications and future directions. Overall, the findings support loading plus ~5 g/day as a pragmatic, effective regimen for muscle and strength gains when combined with resistance training. The absence of bone effects at low doses (1–3 g/day) should not be generalized to other organ systems. Future trials in postmenopausal women should randomize across higher dosing strata (e.g. ≥0.1–0.16 g/kg/day), prespecify nonmuscle endpoints (e.g. cognition, vascular function), and include timing comparisons (daily vs training-day dosing). None of the included studies compared doses head-to-head in women.

Hormone Interactions. We aimed to examine the role of HRT but found insufficient data. Estrogen therapy preserves muscle and bone to some extent; if creatine primarily benefits those with low estrogen, larger effects might occur in non-HRT users. Conversely, if estrogen improves training capacity, it may synergize with creatine. This remains an open question. Future RCTs should stratify or target HRT users vs nonusers to test for differential responses. A recent narrative review suggests creatine could be beneficial during the menopausal transition, when estrogen decline accelerates muscle loss [9]; however, direct evidence is lacking. Our review underscores the need for such targeted research.

Practical Implications: Creatine monohydrate is a practical adjunct to resistance training in women in their 50s–70s. Regimens that use an initial loading phase (~20 g/day for 5–7 days) followed by ~5 g/day, or ~5 g/day without loading, yield modest but clinically meaningful gains in lean mass and strength compared with training alone in this age group. Bone mineral density should not be expected to improve with low-to-moderate dosing without a robust *mechanical* loading stimulus from exercise.

For neurological outcomes, emerging evidence suggests that sustained dosing of ~10 g/day (typically 2 × 5 g/day) may confer greater benefit than lower daily intakes. Although outside the primary focus of the included trials, such protocols can be considered when cognition is a target outcome, preferably within supervised programs and using third-party–tested creatine monohydrate [6]. Across randomized trials in postmenopausal women, supported by large cross-trial safety analyzes, creatine monohydrate has not been associated with a higher incidence of adverse events versus placebo, nor with dehydration or impaired renal function. These conclusions align with position-stand recommendations affirming the safety of creatine monohydrate in healthy adults and clinical cohorts [37].

For bone health, current evidence does not support creatine alone as an osteoporosis intervention. Exercise, especially resistance and high-impact training- remains fundamental; creatine may be added for its muscle benefits. Our findings reinforce that creatine's benefits emerge when paired with exercise.

Dose and regimen advice: A typical loading protocol is 20 g/day for 5 days (four divided doses), followed by 3–5 g/day maintenance. Alternatively, starting at ~5 g/day reaches saturation in ~3–4 weeks, which is acceptable for long-term use and may reduce initial GI upset. Because older adults may have greater GI sensitivity, a slow ramp-up or splitting doses with meals and adequate fluid can help.

Safety reassurance: Concerns about kidney strain, dehydration, or adverse weight gain are not supported by high-quality evidence. A comprehensive 2025 analysis of 685 clinical trials reported no excess total side effects with creatine versus placebo and no signal for renal- or hydration-related harms, corroborating long-standing position-stand conclusions on safety. The modest weight increases sometimes observed reflect lean tissue and intracellular water, not fat mass; in older women undertaking resistance training, creatine increased fat-free/muscle mass without increases in body fat [6,12,25,38].

Limitations: This review synthesized seven randomized controlled trials in postmenopausal women; the modest number of studies limits the precision of pooled estimates and the [25] ability to examine moderators in depth. Clinical heterogeneity was present across dosing strategies (1–5 g/day vs weight-based regimens; with/without loading), intervention length (3–24 months), and training paradigms (supervised resistance training vs no structured exercise), as well as in outcome ascertainment (e.g. 1RM modality, appendicular vs total lean mass, bone geometry vs areal BMD). Some trials reported completer-only outcomes or lacked change-score variances, which constrained meta-analytic inclusion and sensitivity analyzes. Although visual inspection of the funnel plot suggested symmetry and Egger's test was not significant (*p* = 0.40), publication bias cannot be definitively excluded given the small evidence base. Notably, at least two included trials reported negative findings (no between-group differences) and were nevertheless published, indicating that nonsignificant results do reach the literature; however, selective nonpublication elsewhere remains possible. Generalizability is further limited by predominantly healthy samples (few with overt comorbidities), limited racial/ethnic reporting, and sparse head-to-head evaluations of timing or higher-dose protocols designed for nonmuscle endpoints (e.g. cognition).

Future Research: Given the small and heterogeneous evidence base, future multicenter RCTs with standardized outcomes are needed in postmenopausal women. Specifically, well-designed RCTs in perimenopausal women (e.g. late 40 s) could determine whether initiating creatine during the menopausal transition attenuates the typical spike in fat gain and muscle loss in that period. Another priority is to test creatine alongside complementary interventions (e.g. vitamin D or protein) to assess whether additive effects occur in older women. One excluded study ([24] in men) suggested protein did not add to creatine, but this has not been thoroughly evaluated in women. Finally, given interest in sarcopenia prevention, trials in more frail populations (e.g. women > 75 or with sarcopenic obesity) would be valuable; creatine may help individuals who struggle to perform high-intensity training by improving training tolerance.

### 4.1. Practical applications

For sports nutritionists and coaches working with midlife and older female clients, the findings translate into the following guidance. Recommend creatine supplementation as part of a comprehensive training program for postmenopausal women aiming to improve strength or muscle mass. A typical regimen is a 5–7-day loading phase of ~20 g/day (split into four doses) followed by 3–5 g/day maintenance. If loading is contraindicated because of GI discomfort, starting at 3–5 g/day is effective, reaching saturation over several weeks. Emphasize that creatine is not a substitute for exercise. Encourage resistance training at least 2–3 times per week, as the benefits of creatine manifest when combined with progressive overload (e.g. weightlifting or resistance band exercises). Practically, an older woman engaged in resistance training may see slightly greater strength gains with creatine, for example, lifting an additional 5–10 lbs or performing 1–2 more repetitions at a given load, which, accumulated over months, can yield noticeable functional improvements.

## 5. Conclusions

For postmenopausal women engaged in resistance training, creatine monohydrate at doses ≥ 5 g·day⁻¹ or after a brief loading phase yields small-to-moderate strength gains and modest increases in lean mass over 8–24 weeks. Safety outcomes were comparable to placebo, and transient elevations in serum creatinine reflect normal creatine metabolism rather than impaired renal function. By contrast, current evidence does not support creatine monotherapy for preventing bone loss or improving functional capacity without structured exercise. Overall, creatine is safe, inexpensive, and practical, making it a valuable component of comprehensive strategies for healthy aging. This review addresses a notable gap by focusing on midlife and older women. It confirms that postmenopausal women derive measurable benefits from creatine supplementation, particularly when it is integrated with exercise-based programs aimed at mitigating sarcopenia and maintaining functional independence.

## Supplementary Material

Supplementary material Creatine_OSF_Master_Cleaned

Supplementary material Supplementary_Materials_Creatine_Postmenopausals

## Data Availability

The deidentified analysis dataset (cleaned_dataset.csv), Python analysis code (analysis_code.py and notebooks/CreatineMenopause_Meta.ipynb), figure scripts, and output tables/figures are provided in the Supplementary Materials and on our public repository at https://osf.io/bvtrz/. An environment file (requirements.txt) and a reproducibility log CREATINE_OSF_Master) are included to enable exact replication.
