# Supplementary material for: Creatine monohydrate for lean mass, strength, and bone density in postmenopausal women: a systematic review and meta-analysis
Source: J Int Soc Sports Nutr. 2026 May 16;23(1):2668435. doi: 10.1080/15502783.2026.2668435 (PMC13182165; doi:10.1080/15502783.2026.2668435)
Supplement: Supplementary material — Supplementary_Materials_Creatine_Postmenopausals [file RSSN_A_2668435_SM3050.docx]

**SUPPLEMENTARY MATERIALS**

**Contents of this Supplementary File:**

• Table S1 — Full database search strategies

• Appendix A — Post-hoc review protocol (PICOS + analysis plan)

• Table S2 — Summary of Findings (GRADE evidence table)

• Table S3 — Studies excluded at full-text with reasons

• Table S4 — Data dictionary for the linked OSF Excel dataset

*OSF public repository: https://osf.io/bvtrz/*

# Table S1. Full Database Search Strategies

Searches were conducted on 28 August 2025 across seven databases. Boolean operators and controlled-vocabulary terms were adapted for each interface. No language restrictions were applied; publication dates were restricted to 2000–2025 to ensure modern DXA methodology. All queries were peer-reviewed by a research librarian.

| **Database** | **Search Query / Strategy** |
| --- | --- |
| **MEDLINE (PubMed)** | ("creatine"[MeSH Terms] OR "creatine"[tw] OR "creatine monohydrate"[tw] OR "phosphocreatine"[tw]) AND ("postmenopausal"[tw] OR "post-menopause"[tw] OR "postmenopause"[MeSH Terms] OR "menopause"[MeSH Terms] OR "older women"[tw] OR "peri-menopausal"[tw]) AND ("randomized controlled trial"[pt] OR "placebo-controlled"[tw] OR "random*"[tw] OR "trial"[tw]) AND "humans"[MeSH Terms] |
| **Embase** | ('creatine'/exp OR creatine OR 'creatine monohydrate' OR phosphocreatine) AND ('postmenopause'/exp OR postmenopausal OR 'post-menopause' OR menopause OR 'older women' OR 'perimenopause') AND ('randomized controlled trial'/exp OR 'placebo controlled study'/exp OR random* OR trial) AND [humans]/lim |
| **Scopus** | TITLE-ABS-KEY(creatine OR "creatine monohydrate" OR phosphocreatine) AND TITLE-ABS-KEY(postmenopausal OR "post-menopause" OR menopause OR "older women" OR "peri-menopausal") AND TITLE-ABS-KEY(random* OR "placebo-controlled" OR trial OR RCT) |
| **Web of Science Core Collection** | TS=(creatine OR "creatine monohydrate" OR phosphocreatine) AND TS=(postmenopausal OR "post-menopause" OR menopause OR "older women" OR "peri-menopausal") AND TS=(random* OR "placebo-controlled" OR trial) |
| **SPORTDiscus** | (creatine OR "creatine monohydrate" OR phosphocreatine) AND (postmenopausal OR "post-menopause" OR menopause OR "older women") AND (random* OR "placebo-controlled" OR trial) |
| **Cochrane CENTRAL** | [mh creatine] OR creatine OR "creatine monohydrate" AND [mh postmenopause] OR postmenopausal OR "older women" OR menopause — filtered to Trials |
| **ClinicalTrials.gov & WHO ICTRP** | Condition: menopause OR postmenopause; Intervention: creatine; Study type: Interventional (RCT); Status: Any |

*Abbreviations: MeSH, Medical Subject Headings; tw, text word; pt, publication type; RCT, randomised controlled trial; ICTRP, International Clinical Trials Registry Platform.*

# Appendix A. Post-hoc Review Protocol

This protocol was drafted retrospectively, as the review was not prospectively registered. It describes the pre-specified criteria and analysis plan as agreed among authors prior to data extraction.

## A.1 PICOS Eligibility Criteria

Population: Women who are peri-menopausal (in transition per STRAW+10 staging) or postmenopausal (natural menopause, ≥40–45 years; >12 months amenorrhea or FSH confirmation). Studies exclusively in surgically induced menopause or severe comorbid conditions (e.g., active malignancy) were excluded.

Intervention: Creatine monohydrate supplementation, any dosing strategy (loading or non-loading), with or without co-interventions (e.g., resistance training) applied equally to all groups.

Comparator: Placebo or non-caloric control, matched for appearance, taste, and administration route where possible.

Outcomes (primary): (1) DXA-derived lean body mass or appendicular lean mass (kg); (2) maximal dynamic strength assessed by 1-repetition maximum (1RM) or equivalent.

Outcomes (secondary): Bone mineral density (DXA; spine, hip, whole-body); bone turnover markers (CTX, P1NP, osteocalcin); physical performance (Timed Up-and-Go, chair-stand, 6MWT); body composition (fat mass, total body weight); adverse events; clinical laboratory markers (renal: creatinine, eGFR; hepatic: AST, ALT; haematological).

Study design: Randomised controlled trials (parallel-group or crossover). Non-randomised studies, systematic reviews, and animal studies were excluded.

Time frame: Publications from 2000 to August 2025.

## A.2 Data Extraction

A standardised extraction form (Microsoft Excel) was used. Two independent reviewers extracted: author, year, country; participant demographics; intervention details (dose, loading, duration, timing, co-supplements); comparator; supervised resistance training details; and all outcomes (mean ± SD or change scores). Intraclass correlation for extraction agreement was ≥0.95. Author correspondence was attempted when variance data were missing; one clarification was received.

## A.3 Risk of Bias Assessment

The Cochrane Risk of Bias 2 (RoB 2) tool was applied independently by two reviewers across five domains: (1) randomisation process, (2) deviations from intended interventions, (3) missing outcome data, (4) measurement of the outcome, (5) selection of the reported result. Overall ratings: Low, Some Concerns, or High. Disagreements were resolved by consensus (third reviewer if needed).

## A.4 Statistical Analysis Plan

Effect measure: Mean difference (MD) in original units (kg) for lean mass and 1RM strength. Standardised mean difference (Hedges' g) was computed for cross-unit pooling where necessary.

Heterogeneity variance estimator: Paule–Mandel (PM) τ² with Hartung–Knapp–Sidik–Jonkman (HKSJ) confidence interval adjustment.

Heterogeneity statistics: Cochran Q (p-threshold 0.10); I² (≤40% low, 50–75% moderate, >75% high); τ²; 95% prediction interval (PI).

Subgroups: Resistance training (RT vs no RT) — pre-specified. Subgroup interaction tested via mixed-effects meta-regression.

Meta-regression: Exploratory univariable regression on daily creatine dose (g/day) for lean mass — conducted only if k ≥ 5 studies (threshold met).

Sensitivity analyses: Leave-one-out; Baujat plot; change in correlation assumption (r = 0.3, 0.5, 0.7) for imputed change-score SDs.

Publication bias: Visual funnel plot inspection; Egger's regression test (p < 0.40 threshold applied given low power); trim-and-fill.

Certainty: GRADE (Grading of Recommendations, Assessment, Development and Evaluations) per outcome.

Software: R 4.3 with metafor package (v3.8) and Python 3.11 (pandas, matplotlib, scipy). Code and datasets are available at https://osf.io/bvtrz/.

# Table S2. Summary of Findings (GRADE Evidence Profile)

Population: Postmenopausal women. Intervention: Creatine monohydrate (any dose). Comparator: Placebo. Setting: Community / research-clinic; 12–104 weeks.

| **Outcome** | **No. of Studies (participants)** | **Study Design** | **Risk of Bias** | **Inconsistency** | **Imprecision** | **Effect Estimate (95% CI) & Certainty** |
| --- | --- | --- | --- | --- | --- | --- |
| Lean mass (kg) | 5 RCTs (n = 338) | RCT | Some concerns | Low (I² = 25%) | Moderate | MD +0.37 kg (+0.05 to +0.69); MODERATE certainty |
| Leg-press 1RM strength (kg) | 3 RCTs (n = 111) | RCT | Some concerns | None (I² = 0%) | Moderate | MD +7.5 kg (+2.2 to +12.8); MODERATE certainty |
| Bone mineral density – femoral neck (g/cm²) | 3 RCTs | RCT | Low risk / Some concerns | Moderate | Moderate | MD ≈ 0.00 g/cm² (95% CI spans null); HIGH certainty for no important benefit |
| Physical function (TUG, walk tests) | 2–3 RCTs | RCT | Some concerns | Moderate | Serious | No significant between-group difference; LOW certainty |
| Adverse events / Safety | 7 RCTs (685 trial cross-trial analysis) | RCT + large safety dataset | Low risk | None | None | No excess AEs (creatine 4.60% vs placebo 4.21%, p = 0.828); HIGH certainty for safety |

*CI, confidence interval; MD, mean difference; RCT, randomised controlled trial; RoB, risk of bias; TUG, Timed Up-and-Go.*

*GRADE certainty: HIGH = further research very unlikely to change confidence in effect estimate; MODERATE = further research likely to have important impact on the estimate; LOW = further research very likely to have important impact; VERY LOW = very uncertain about the estimate.*

# Table S3. Studies Excluded at Full-Text Review with Reasons

Fifty-two full-text articles were reviewed; seven were included in the primary meta-analysis. Additional qualitative studies are noted separately in Table 2 of the main manuscript. The table below lists the most commonly excluded study types to illustrate the exclusion process.

| **Study** | **Population / Design** | **Key Reason for Exclusion** | **Notes** |
| --- | --- | --- | --- |
| Cañete et al., 2006 (JSCR) | Elderly women (~67 y), DB-RCT, 7 days, 0.3 g/kg/d loading, no RT | Acute ≤7-day trial; no RT; menopausal status not confirmed | Included in qualitative narrative only |
| Gotshalk et al., 2008 (EJAP) | Older women 58–71 y, DB-RCT, 7 days, 0.3 g/kg/d loading, no RT | Acute ≤7-day trial; no RT; menopausal status not confirmed | Included in qualitative narrative only |
| Alves et al., 2013 (PLOS ONE) | Older women (60–80 y), DB 4-arm factorial RCT, 24 wk, 20 g/d load → 5 g/d; RT or no RT | No DXA lean mass outcome; menopausal status not confirmed; no placebo for creatine-only arm | Strength sensitivity analysis candidate |
| Evans et al., 2017 (Nutrition & Metabolism) | Healthy older adults (mixed sex), DB-RCT, 8 wk, creatine + L-carnitine + leucine | Multi-ingredient supplement, not creatine monohydrate alone; mixed sex without disaggregated female data | Background reference only |
| Neves et al., 2011 | 26 postmenopausal females, DB-RCT, 12 wk, 5 g/d + RT | Safety sub-study of another included trial; renal outcomes only — no lean mass or strength primary data | Renal safety narrative |
| Johannsmeyer et al. (cited in Candow 2015) | Mixed-sex older adults, no sex-disaggregated data | No separate postmenopausal female data | Background reference only |
| Roschel et al., 2021 | 200 participants (77% women, 23% men), 16 wk, CrM + whey vs placebo | Mixed-sex; creatine combined with whey (not isolated CrM); frailty population; postmenopausal data not disaggregated | Background reference (frailty) |

*DB-RCT, double-blind randomised controlled trial; RT, resistance training; DXA, dual-energy X-ray absorptiometry; CrM, creatine monohydrate.*

# Table S4. Data Dictionary — OSF Excel Supplementary Dataset

The linked Excel workbook (Creatine_OSF_Master_Cleaned.xlsx) contains six sheets. Variable definitions for the primary sheets (Included_RCTs, Final_Table_Postmenopausal, Safety_Data_Female_Older, Final_Table_Master) are defined below. Numerical codes in Safety_Data_Female_Older use the same variable names.

| **Variable Name** | **Type / Values** | **Description** |
| --- | --- | --- |
| Study | Text (full citation) | Full bibliographic reference for the included study |
| Author | Text (short label) | First author name and year (e.g., Aguiar et al. (2013)) |
| Participants | Text | Number and description of participants enrolled |
| Design | Categorical: RDBP, RPDB, RDBPC, OL, CS | Study design abbreviation |
| Demographics | Text | Age and weight by group (creatine and placebo) |
| Duration | Numeric (days) | Total intervention duration in days |
| Loading_Days | Numeric (days) or '–' | Number of days on loading dose; dash if no loading phase |
| Non-Loading_Days | Numeric (days) | Number of days on maintenance/non-loading dose |
| Findings | Text (narrative) | Summary of primary and secondary outcome findings |
| Side_Effects | Text / 'None reported' | Adverse events reported in the creatine or placebo arms |
| Sex | Categorical: Female, Male, Combined Cohort, Unspecified | Sex of participants |
| Age_Category | Categorical | Children/Adolescents (<18 y), Young Adults (18–45 y), Middle-Age (45–65 y), Older Adults (>65 y) |
| Population_Category | Categorical | Training status: Untrained, Trained, Athlete, Recrtationally Active, Clinical–Physical/Health, etc. |
| Health_Status | Categorical | Apparently Healthy vs clinical sub-categories |
| Clinical_Population_Category | Categorical | Specific clinical condition if applicable |
| Type_Of_Creatine_Used | Categorical | Creatine Monohydrate or Other |
| CrM_Vs_All_Other_Creatine_Forms | Categorical: Creatine Monohydrate, Other | Whether the supplement was pure CrM |
| Year | Numeric (4-digit) | Publication year |
| Included_In_MA | Categorical: Yes / No | Whether study contributed data to the primary meta-analysis |
| Population_Relevance | Categorical | Postmenopausal/Older Women, Middle-Age/Older (Potentially relevant), or Younger/Children (Background only) |

*Sheet descriptions: (1) Included_RCTs — the 7 RCTs included in the primary meta-analysis; (2) Final_Table_Postmenopausal — all 52 female creatine studies in the postmenopausal/women catalogue with relevance flags; (3) Safety_Data_Female_Older — safety data filtered to female, middle-age or older participants; (4) Final_Table_Master — master catalogue of all creatine studies (background reference); (5) Data_Dictionary — variable definitions; (6) Variable_Descriptions_Raw — original variable/label mapping.*

*OSF public repository (data, code, figures):* [*https://osf.io/bvtrz/*](https://osf.io/bvtrz/)

In Strength,

Sia
